# Transcriptome, Biochemical and Phenotypic Analysis of the Effects of a Precision Engineered Biostimulant for Inducing Salinity Stress Tolerance in Tomato

**DOI:** 10.3390/ijms24086988

**Published:** 2023-04-10

**Authors:** Elomofe Ikuyinminu, Oscar Goñi, Łukasz Łangowski, Shane O’Connell

**Affiliations:** 1 Plant Biostimulant Group, Shannon Applied Biotechnology Centre, Munster Technological University-Tralee (South Campus), Clash, V92 CX88 Tralee, Co. Kerry, Ireland; 2 Brandon Bioscience, V92 N6C8 Tralee, Co. Kerry, Ireland

**Keywords:** plant biostimulants, protein hydrolysate, *Ascophyllum nodosum* extract, irrigation salinity stress, osmotic adjustment, ion homeostasis, transcriptome, *Solanum lycopersicum* L., yield

## Abstract

Salinity stress is a major problem affecting plant growth and crop productivity. While plant biostimulants have been reported to be an effective solution to tackle salinity stress in different crops, the key genes and metabolic pathways involved in these tolerance processes remain unclear. This study focused on integrating phenotypic, physiological, biochemical and transcriptome data obtained from different tissues of *Solanum lycopersicum* L. plants (cv. Micro-Tom) subjected to a saline irrigation water program for 61 days (EC: 5.8 dS/m) and treated with a combination of protein hydrolysate and *Ascophyllum nodosum*-derived biostimulant, namely PSI-475. The biostimulant application was associated with the maintenance of higher K^+^/Na^+^ ratios in both young leaf and root tissue and the overexpression of transporter genes related to ion homeostasis (e.g., *NHX4*, *HKT1;2*). A more efficient osmotic adjustment was characterized by a significant increase in relative water content (RWC), which most likely was associated with osmolyte accumulation and upregulation of genes related to aquaporins (e.g., *PIP2.1*, *TIP2.1*). A higher content of photosynthetic pigments (+19.8% to +27.5%), increased expression of genes involved in photosynthetic efficiency and chlorophyll biosynthesis (e.g., *LHC*, *PORC*) and enhanced primary carbon and nitrogen metabolic mechanisms were observed, leading to a higher fruit yield and fruit number (47.5% and 32.5%, respectively). Overall, it can be concluded that the precision engineered PSI-475 biostimulant can provide long-term protective effects on salinity stressed tomato plants through a well-defined mode of action in different plant tissues.

## 1. Introduction

Salinity is a major adverse factor responsible for productivity loss of important high value vegetable crops worldwide. This problem is gradually increasing in arid and semi-arid regions where irrigated agriculture is fundamental to produce high crop yields [1]. The presence of a high concentration of salt in the root zone, Na^+^ accumulation, causes significant changes in several phenotypical, physiological, biochemical, and molecular parameters of plants. These changes are dependent on the severity (measured through electric conductivity (EC) of soil or irrigation water) and duration of salinity stress. Increasing salinity eventually decreases crop production to the point of it not being economically viable or crops fail. While different temporal and spatial salinity stress models have been proposed, it is widely accepted that plants are subjected to osmotic and ion toxicity stresses, whose adverse effects can overlap [2,3]. 

To withstand the adverse effects of salinity stress, plants have developed several tolerance mechanisms articulated at a phenotypical, physiological, biochemical, and molecular level. Some of these tolerance mechanisms include the modification of the expression level of salt-responsive genes which have downstream effects on osmotic stress tolerance, ion homeostasis, reactive oxygen species (ROS) scavenging, protein and membrane protection, photosynthetic efficiency, carbon (C) and nitrogen (N) metabolism and stress signalling pathways [3,4,5]. To preserve cell volume and turgor, plants exposed to salinity stress can change cell wall elasticity, improve water uptake and distribution through enhanced activities of aquaporins and accumulation of osmolytes in the cytoplasm [6,7]. Activation of ion detoxification mechanisms such as Na^+^ accumulation in root tissue, reduced Na^+^ translocation to aboveground plant organs, Na^+^ compartmentalization at a vacuolar level and/or improved K^+^ uptake and transport is facilitated through higher abundance and activity of ion channels and transporter families such as salt overly sensitive (SOS), high-affinity potassium transporter (HKT), sodium/hydrogen antiporters (NHXs), potassium transporters (AKT) and high-affinity K^+^ transporter (HAK) [8,9]. Likewise, cell oxidative damage on protein complexes and cell membranes can be ameliorated through the protective action of molecular chaperones such as heat shock proteins (HSPs) and late embryogenesis abundant (LEA) proteins [10,11]. Other activated salinity stress tolerance mechanisms are related to increased gene expression and activity of enzymes related to photosynthetic efficiency, biosynthesis of photosynthetic pigments, carbohydrates, and amino acids [12,13,14,15]. Moreover, these biological responses are regulated by numerous transcription factors and endogenous phytohormones [16,17].

RNA sequencing (RNA-Seq) technology is an effective tool for measuring multiple gene expression changes in plants subjected to different salinity stress conditions. Plant biostimulants provide a technological agronomic solution to the increasing global demand for high crop productivity while using environmentally friendly inputs [18,19]. In addition to the beneficial role of commercial *Ascophyllum nodosum* extracts (ANEs) and protein hydrolysate biostimulants (PHBs) on plant growth, crop yield, NUE and crop quality traits, there are several examples of their ability to stimulate responses in plants and crops to cope with abiotic stresses [20,21]. Although transcriptome studies have also been used to evaluate the effect of ANEs and PHBs on plants [22,23,24,25,26,27], most of the effects of ANEs and PHBs at the molecular level have been developed in plants growing under unstressed conditions, short termed and linked to phytohormone, antioxidant and nutrient-related activities [28]. Therefore, the mode of action of ANEs and PHBs in enhancing plant tolerance to abiotic stresses remains relatively unexplored, especially in terms of integrating phenotypic, physiological, biochemical and transcriptome information. 

Previously, we showed that a precision-engineered biostimulant (PSI-475) can alleviate the negative effects of saline irrigation water on tomato productivity and quality [29]. However, the detailed response mechanism following the stimulation remained to be revealed. In the current study, we have elucidated the detailed mode of action of PSI-475 in enhancing salinity stress tolerance in different tissues of tomato plants (cv. Micro-Tom) subjected to a long saline irrigation program until the fruit harvest stage. A transcriptome study using next generation sequencing of RNA extracted from young leaf and root tissue was conducted to determine the molecular markers and salinity stress tolerance signalling pathways induced by PSI-472. The impact of the application rate of PSI-475 was also determined using plant phenotypic and fruit yield parameters. The effect of PSI-475 on physiological markers and metabolite partitioning in root, stem, and leaf tissues at the fruit filling stage were also assessed. The results were interpreted in context of the transcriptome analysis, providing further insight into understanding how the precision-engineered biostimulant PSI-475 can help growers to mitigate the adverse influence of saline irrigation water in order to enhance crop productivity.

## 2. Results

### 2.1. Effect of Salinity and PSI-475 Rate on Tomato Phenotypic and Fruit Yield Parameters

**Figure 1:**
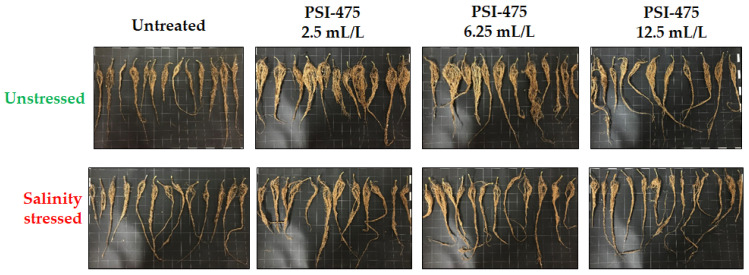
Effect of salinity and PSI-475 dosage rate on tomato root growth. Roots shown in the pictures were sampled from 10 to 12 different plants per treatment and condition.

To illustrate the effect of salinity on tomato growth and development, two biomass traits (plant biomass corresponding to all aboveground organs except fruits and root biomass) and two fruit yield parameters (fruit number and fruit yield per plant) were evaluated at the end of the tomato plant trials developed under unstressed and salinity stressed conditions after applying three different concentrations of PSI-475 (Table 1). The application of a saline irrigation water program for 61 days (EC: 5.7 dS/m) negatively affected plant and root biomass (*p* ≤ 0.001), decreasing their average values by 23.3 and 15.0%, respectively. The application of PSI-475 at three different rates had a statistically significant positive effect on both plant and root biomass compared to untreated plants under either unstressed or stressed conditions. While root biomass increased between 83.0% and 96.5% regardless of PSI-475 rate (Figure 1), spraying 2.5 mL/L or 6.25 mL/L of biostimulant led to the highest benefits on plant biomass (+12.7 and +12.9%, respectively).

**Figure 2:**
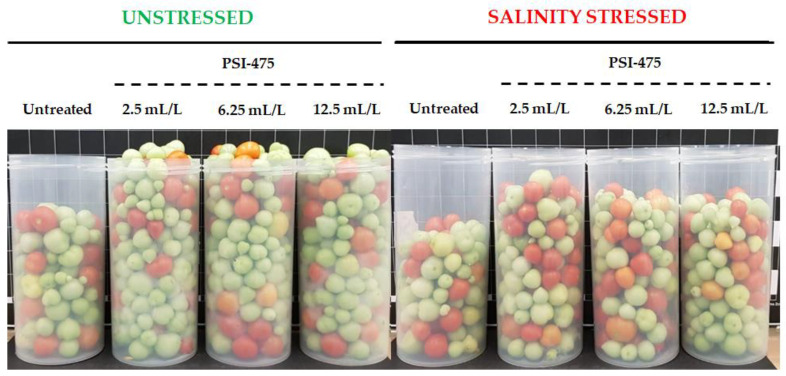
Effect of salinity and PSI-475 dosage rate on tomato fruit number and yield. Fruits shown in the pictures were harvested from 11 plants per treatment and condition.

As observed in Figure 2, fruit yield and average fruit number were also reduced in salinity stressed untreated plants (−26.3% and −13%, respectively). PSI-475 was effective in increasing both fruit yield and fruit number at the lowest dose applied (2.5 mL/L) under both unstressed and stressed conditions (+47.5% and +32.5% on average, respectively) (Table 1). Dose rate analysis clearly indicated that the lowest rate of PSI-475 (2.5 mL/L) was the most beneficial in terms of phenotypical and yield benefits. Therefore, effects of this concentration of biostimulant were further assessed and described at physiological, biochemical, and molecular level in unstressed and salinity stressed plants. 

### 2.2. Effect of Salinity and PSI-475 on Tomato Leaf RWC

**Figure 3:**
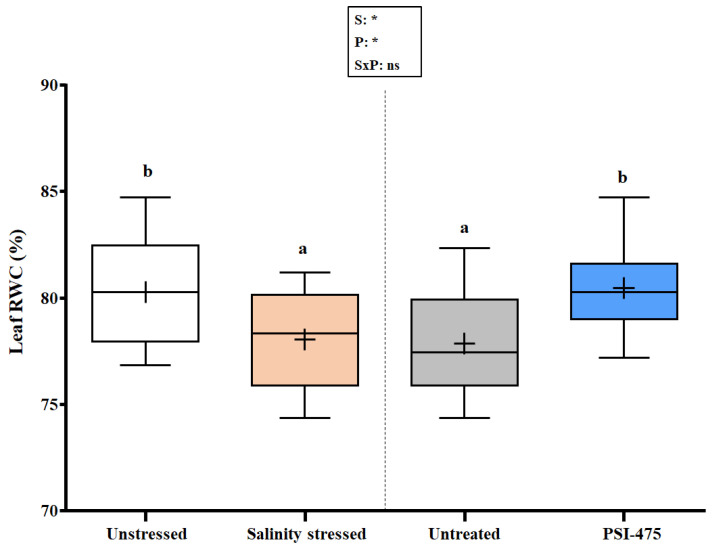
Effect of salinity stress and PSI-475 on tomato young leaf RWC. Since there was no significant S × P interaction, the effect of condition and PSI-475 treatment was evaluated separately, comparing the respective means. Different letters indicate statistically significant differences for *p* ≤ 0.05 based on *t*-test (condition and treatment). The vertical dashed line was used to visually separate the evaluation of the effect of condition and PSI-475 treatment. The horizontal line through the box and the cross represents the median and mean value, respectively. Source of variance was included in the figure (ns, *: non-significant or significant at *p* ≤ 0.05, respectively).

To determine the effect of osmotic stress on tomato plants at harvest time, RWC in young leaf tissue was selected as a physiological marker in both untreated and treated plants with PSI-475 applied at 2.5 mL/L. The two-way ANOVA showed that both salinity stress and PSI-475 treatment were statistically significant, *p* = 0.026 and 0.011, respectively, with no significant interaction between both factors (S × P) (Figure 3). There was a significant decrease of RWC between unstressed (80.27%) and salinity stressed (76.05%) conditions (*p* = 0.026). The application of PSI-475 also positively affected young leaf RWC, by increasing its average value by 3.3% compared to untreated plants in both unstressed and stressed plants.

### 2.3. Effect of Salinity and PSI-475 on Sodium and Potassium Content in Different Tomato Tissues

The application of a 61-day saline irrigation program using 50 mM NaCl (5.8 dS/m) from the early flowering stage did have a significant effect on growth medium EC at harvest time, reaching a value that was 6-fold higher than that measured in growth medium from unstressed plants (15.82 versus 2.55 dS/m, respectively) and confirming the strong salinity stress present on the root zone area.

The analysis of Na^+^ content showed a clear accumulation pattern in all tissues of salinity stressed plants at harvest time, leading to increased values that ranged between 2.3-fold in root tissue to 49.1-fold in young leaf tissue (Table 2). Na^+^ content was also significantly affected by PSI-475 in different tissues from salinity stressed plants. While root and lower stem tissue in treated stressed plants increased their Na^+^ content by 31.7% and 25.7%, respectively, measured levels in upper stem and young leaf tissues recorded a moderate decrease (19.0% and 26.2%, respectively). However, there was no statistically significant difference in old leaf Na^+^ content between treated and untreated salinity stressed plants. 

The significant disruption of K^+^ homeostasis by salinity stress in all plant tissues was evident, decreasing between 11.1% and 22.9% between stressed and unstressed untreated plants (Table 3). While PSI-475 increased K^+^ content in root tissues, the statistically significant interaction between factors (S × P) indicated that this accumulation was more pronounced in unstressed than stressed plants (2.2-fold vs. 1.9-fold, respectively). However, a differential effect was observed in the lower stem of PSI-475 treated plants, where K^+^ content decreased by 15.2% in unstressed plants but increased by 11.0% in stressed plants. Interestingly, in both upper stem and young leaf tissues, there was a minor statistically significant decrease of K^+^ content under both unstressed and salinity stressed conditions (10.3% and 10%, respectively).

When the well-established salinity stress tolerance marker K^+^/Na^+^ ratio was calculated, it was evident that there was a significant decrease in all tested tissues, ranging from 3.2-fold to 77.8-fold reduction in root and young leaf tissues (Table S1). Two-way ANOVA revealed the interaction between factors (S × P) and, interestingly, PSI-475 had a moderate positive statistically significant effect on this tolerance marker on root and young leaf tissue from stressed plants (increasing K^+^/Na^+^ ratio by 1.3- and 1.2-fold, respectively) and a minor non-statistically significant increase in old leaf tissue (+2.3%). While this tendency was similar in root and young leaf tissue from treated unstressed plants, the old leaf K^+^/Na^+^ ratio decreased by 6.5% once salinity stress was not present.

### 2.4. Effect of Salinity and PSI-475 on Proline and Soluble Sugars Content in Different Tomato Tissues

To better understand the results obtained on RWC in young leaf tissue described above, the accumulation of osmolytes such as proline and soluble sugars (glucose, fructose, and sucrose) was determined in different tissues (root, lower stem, old leaf, upper stem, and young leaf) of plants untreated and treated with PSI-475 growing under unstressed and salinity stressed conditions. As can be observed in Table 4, the two-way ANOVA analysis showed that salinity stress at harvest time had a strong statistically significant effect on the accumulation of proline content in the five sampled tissues (*p* ≤ 0.001), increasing by 2, 5.6, 8.9, 5.6 and 13.1-fold with respect to that measured in unstressed root, lower stem, old leaf, upper stem and young leaf tissue, respectively. There was no statistically significant interaction between factors (S × P) in root tissue and PSI-475 had a general stimulating effect by increasing proline content by 12.7% on average in both unstressed and stressed plants. However, the interaction S × P was statistically significant (*p* ≤ 0.001) in the rest of the aboveground plant organs. This was characterised by similar or lower proline content in stem and old leaf tissues of treated plants growing under unstressed and stressed conditions but a significant accumulation in young leaf of stressed plants treated with PSI-475 (+8.5%) (Table 4).

Sucrose content was quantified by HPAEC-PAD in root, lower stem, old leaf, upper stem, and young leaf tissues of tomato plants. Unlike proline levels, sucrose content was only significantly accumulated by salinity stress in root, old leaf, and young leaf tissue (1.8, 1.9 and 2.3-fold, respectively; *p* ≤ 0.001). An interaction between factors (S × P) was observed for root sucrose content (*p* ≤ 0.001). This interaction was characterised by a higher accumulation of sucrose in treated stressed plants (+95%) than in treated unstressed plants (+81%) compared to their respective controls. PSI-475 also had a statistically significant effect in increasing sucrose content in lower stem, upper stem, and young leaf tissue of both unstressed and stressed plants at harvest time (+64%, +19% and +36%, respectively; *p* ≤ 0.01) Table 5.

Regarding glucose content, salinity significantly increased the amount of this monosaccharide in both leaf tissues by between 2.2 and 3.0-fold but it was reduced in lower stem and upper stem by 30% and 50% (Table S2). Interestingly, PSI-475 application caused a statistically significant glucose increase in all tissues tested under both unstressed and salinity stressed conditions (between 1.2 and 3.2-fold). An interaction between factors (S × P) was observed for lower and upper stem tissues and glucose accumulation was more pronounced in unstressed treated plants (4.9 and 5.6-fold higher than control, respectively).

Fructose content was only significantly affected by salinity in old leaf (+4.7-fold), upper stem (−36%) and young leaf (+1.9-fold) tissues (Table S3). PSI-475 had a similar stimulating effect on this monosaccharide as that observed previously with glucose (between 1.2 and 2.2-fold). The observed interaction between factors (S × P) in root, lower stem, upper stem, and young leaf tissue indicated that this accumulation was also more pronounced in treated unstressed plants (between 2.0 and 4.9-fold higher than control). 

### 2.5. Effect of Salinity and PSI-475 on Tomato Soluble Protein and Photosynthetic Pigments

Two-way ANOVA showed that salinity stress only had a statistically significant effect on soluble protein content in root and old leaf tissue, reducing this parameter by 37.1% and 31.7%, respectively (Table S4). Notably, PSI-475 application stimulated the accumulation of soluble protein content of unstressed and stressed young leaf tissues (+11% and 23.4%, respectively) but it was strongly reduced in stressed old leaf tissues from treated plants compared to control (−42.7%).

The data in Table 6 indicate noticeable adverse effects of salinity stress on photosynthetic pigments (measured as chlorophyll a + b and carotenoids) from old leaf (−32% to −36%) and young leaf (−18% to −16%), confirming that chlorosis symptoms, a key phenotypic trait of salt stress, was present in older leaf organs. Interestingly, PSI-475 application in stressed plants had only a statistically significant effect on young leaf tissue, increasing their chlorophyll (a + b), and carotenoids content by 27.5% and 19.8%, respectively. The same treatment in unstressed young leaf tissue did not affect their carotenoid content.

### 2.6. RNA Sample Characterisation, RNA Sequencing and Mapping

Samples of root and young leaves were collected on the 127th day of tomato growth (late fruit filling stage/early fruit harvest stage) from control plants and plants treated with PSI-475 applied at 2.5 mL/L growing under salinity stressed conditions. Between 21 and 28 million raw reads were generated from 12 cDNA libraries. After filtering the raw data, between 20 and 27 million clean reads were obtained per sample. The base error rate of whole sequencing was very low (0.02%), and the percentage of bases with Q20 and Q30 was higher than 98% and 95%. A significantly elevated percentage of obtained clean reads were successfully mapped to the tomato reference genome assembly SL3.0 in all the samples sequenced (94.95% to 96.93%) (Table S5).

### 2.7. Differentially Expressed Genes (DEGs) in Young Leaf and Root Tomato Tissues

**Figure 4:**
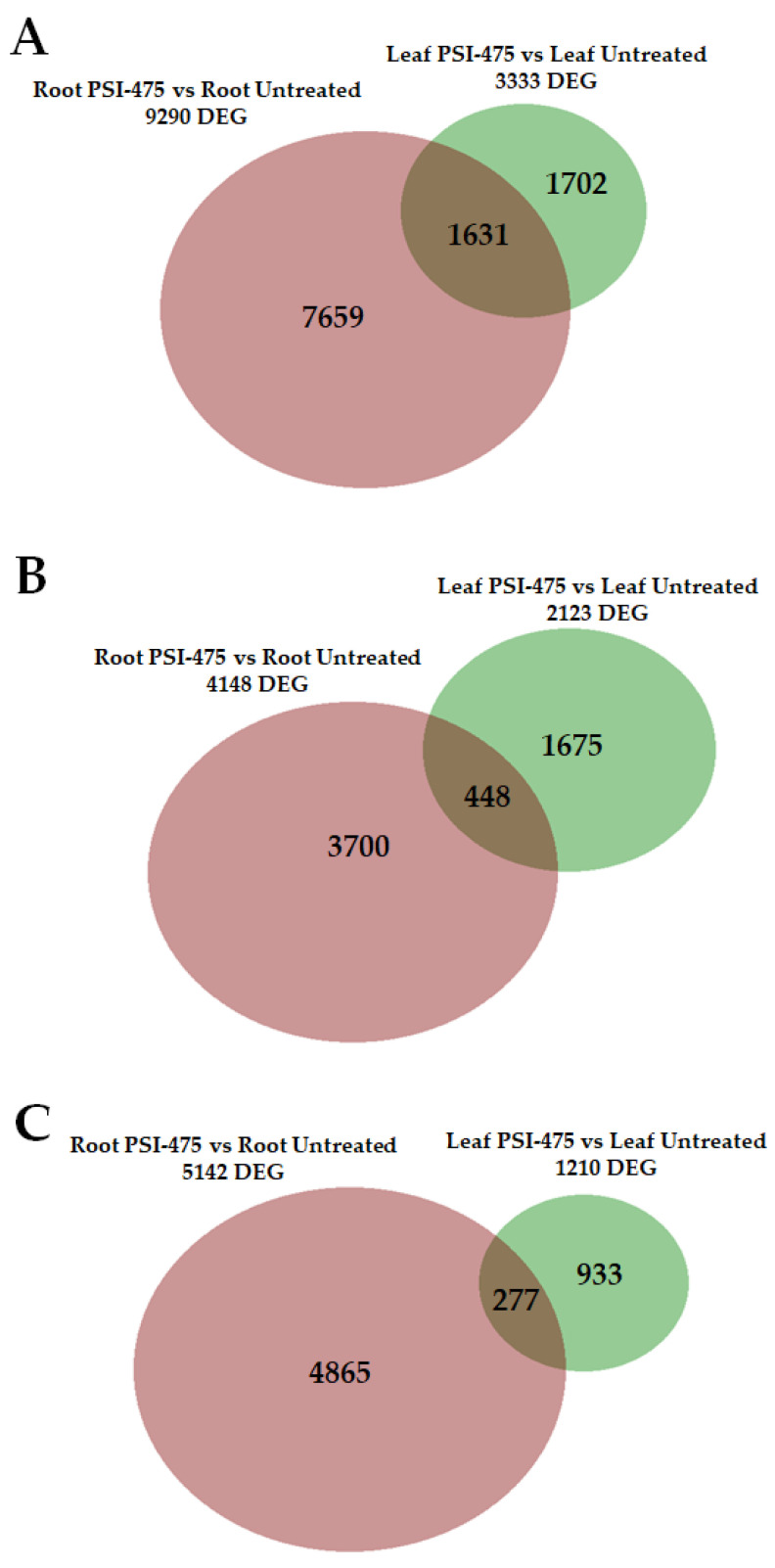
Venn diagram showing the comparison in gene expression of salinity stressed tomato plants treated with PSI-475 in root and young leaf tissue. (**A**) Total DEGs; (**B**) upregulated DEGs; (**C**) downregulated DEGs. Non-overlapping numbers represent the number of genes unique to each tissue. Overlapping numbers represent the number of mutual genes between tissues.

As observed in the Venn diagram analysis, there were more upregulated genes (2123) than downregulated genes (1210) in treated young leaf tissue. However, the opposite was observed in treated roots, with 4148 genes showing an increased expression and 5142 genes having a significantly decreased expression compared to control (Figure 4). There was overlapping in the gene expression response patterns induced by PSI-475 in both analysed tissues of salinity stressed plants. While 7659 and 1702 genes were specifically dysregulated in treated root and young leaf tissue, respectively, 1631 genes were commonly dysregulated (which represents 48.9% of young leaf DEGs) (Figure 4A). Among the genes significant upregulated by PSI-475, 3700 and 1675 genes were unique in treated root and young leaf tissues, respectively, with only 448 genes commonly upregulated in both tissues (Figure 4B). Regarding the downregulated DEGs, 4865, 933 and 277 genes were significantly repressed in the root tissue, leaf tissue and both tissues of PSI-475 treated plants, respectively (Figure 4C).

### 2.8. Functional Classification of Gene Expression Patterns

**Figure 5:**
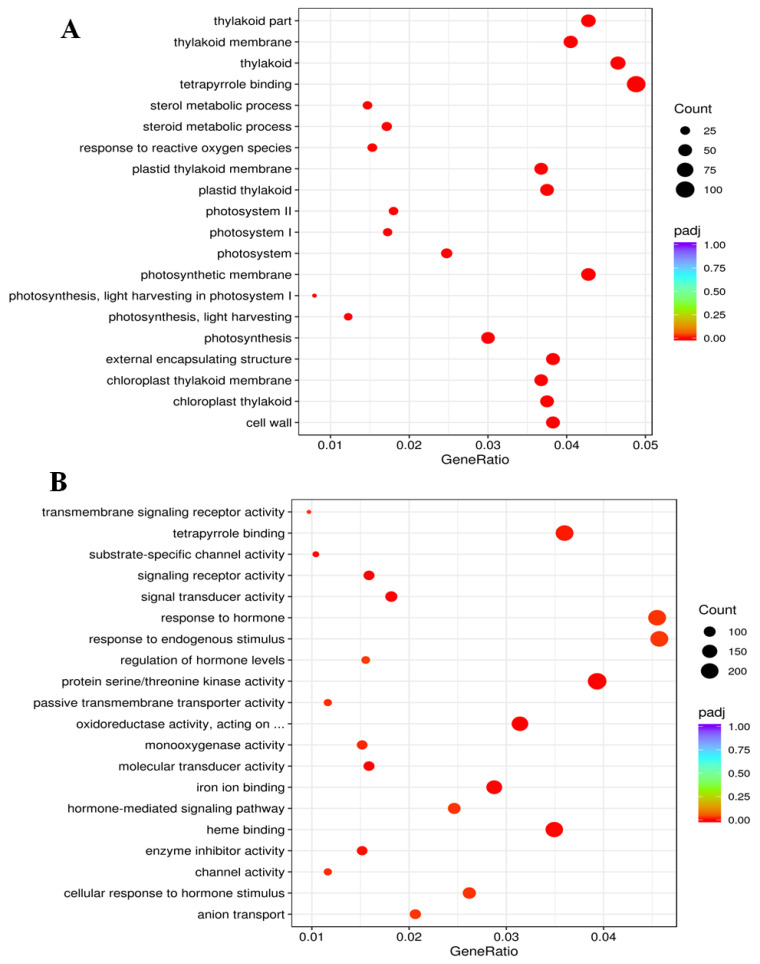
GO enrichment analysis of young leaf and root tissue. A dot diagram was used to represent the largest GO terms in biological processes, molecular functions, and cellular components ontologies in young leaf (**A**) and root (**B**) tissue. The number of DEGs in each GO term is characterized by the size of the circle and colour of the circle represents the adjusted *p*-value.

We annotated the DEGs from treated young leaf and root tissue of tomato plants under salinity stress with biological processes, molecular functions, and cellular components ontologies. In treated young leaf tissue, the top three largest GO terms in the biological process were “photosynthesis”, “response to reactive oxygen species” and “sterol metabolic process”; in the molecular function, “tetrapyrrole binding” was the top largest GO term; and in the cellular component, the top three biggest GO terms were “thylakoid”, “photosynthetic membrane” and “thylakoid part” (Figure 5A). In treated root tissue, the largest three GO terms in the biological process were “response to endogenous stimulus”, “response to hormone”, and “cellular response to hormone stimulus”; in the molecular function, the biggest three GO terms were “protein serine/threonine kinase activity”, “tetrapyrrole binding” and “heme binding”; and no statistically significant GO terms were observed in the cellular component (Figure 5B).

### 2.9. Modulation of Genes Related to Metabolic Pathways

**Solute transport:** There were 134 and 309 DEGs related to this pathway in young leaf and root tissue after applying PSI-475, respectively. Focusing on those DEGs related to either K^+^/Na^+^ homeostasis or osmotic stress tolerance mechanisms, there was a significant dysregulation of genes encoding monovalent cation transporters and aquaporins. Regarding Na^+^ transporters, the vacuolar antiporter *NHX4* (*solyc01g098190.3.1*, FC +2.71) was found to be upregulated in leaf tissue along with four cation/proton antiporters [*CHX15-like* (*solyc06g050690.3.1*, FC +2.46); *CHX15-like* (*solyc08g016160.3.1*, FC +1.97); *CHX18-like* (*solyc08g081810.3.1*, FC +1.54); *CHX20* (*solyc09g010530.3.1*, FC +1.81)]. Several DEGs related to Na^+^ transport were also identified in root tissue of stressed plants treated with PSI-475. The selective HKT1;2 transporter, which is involved in Na^+^ removal from xylem flow, was found to be strongly upregulated in root tissue (*solyc07g014680.3*, FC +11.85). Likewise, three genes encoding cation/proton antiporters were also dysregulated: [*CHX18* (*solyc12g056160.2.1*, FC +3.63); *CHX20* (*solyc09g010530.3.1*, FC +36.74); *CHX28*, (*solyc04g015990.2.1*, FC −13.43)]. K^+^ transporters play an important role in maintaining ion homeostasis when plants are subjected to salinity stress conditions. In total, 6 out 7 DEGs related to K^+^ transport were upregulated in young leaf tissue of stressed plants (*HAK25-like*, *HAK12*, *SKOR-like*, *AKT1*, *TPK1*, and *CHX20*). A higher number of DEGs were found in root tissue, upregulating 10 of them (*HAK11-like, HAK12-like, HAK4-like, KEA2, KAT3-like, AKT2/3, SKOR, TPK1*, and *CHX20*) while 3 genes encoding voltage-gated K^+^ channels were downregulated (*AKT1, TPK3* and *TPK5*). 

Aquaporins, a large family of major intrinsic proteins that form pores in the cell membranes of cytoplasm and vacuoles to facilitate water transport, can play a critical role in ameliorating distorted water uptake and transport induced by salinity stress. In young leaf tissue, 8 out of 9 DEGs identified as aquaporins were upregulated by PSI-475 treatment (*NIP-type, Lsi-1, PIP1;2*, *TIP-like*, *TIP1;3, TIP4;1, TIP2;1,* and *TIP3;1*). Regarding root tissue, while 11 genes encoding different aquaporin classes were upregulated (*Lsi-1*, *PIP-like*, *PIP2;4, PIP1;5, PIP2;1-like, PIP2;1, PIP3;1, TIP*, *aquaporin like*, *TIP1;1,* and *TIP2;1*), 3 DEGs were downregulated (*PIP1;2, TIP4;1,* and *SIP1;3*).

**Photosynthesis:** There were 43 and 4 DEGs upregulated and downregulated, respectively in young leaf tissue after applying PSI-475. Genes involved in photosynthetic efficiency were upregulated in the treated young leaf tissue with respect to control, including those ones encoding light-harvesting complex (LHC) membrane intrinsic proteins from photosystem I (PSI) and photosystem II (PSII) antenna such as *LHCa4* (*solyc06g069730.3.1*, FC +12.27), *LHCa1* (*solyc05g056050.3.1*, FC +12.20), *LHCb1/2/3 (solyc03g005760.1.1*, FC +9.42), *LHCa2* (*solyc10g006230.3.1*, FC +2.64), and *LHCb6* (*solyc01g105050.3.1*, FC +2.61). The upregulation of genes encoding a ferredoxin electron carrier gene (*solyc01g103920.3.1*, FC +2.53) and the CGL160 factor involved in ATP synthase complex assembly (*solyc07g055050.3.1*, FC +4.29) was also observed. Moreover, the overexpression of different genes involved in the Calvin cycle was also significant, such as transcripts encoding the small subunit of RuBisCo (*solyc02g063150.3.1*, FC +1.57), proteins involved in RuBisCo assembly and activity regulation [(*solyc11g069790.2.1*, FC +1.78); (*solyc09g011080.3.1*, FC +3.57)] and glyceraldehyde 3-phosphate dehydrogenase enzyme (*solyc04g082630.3.1*, FC +1.86).

**Coenzyme metabolism:** There were 16 and 2 DEGs upregulated and downregulated in young leaf tissue after applying PSI-475. Most of the DEGs found in this functional category were involved in different tetrapyrrole metabolic pathways, which are critical to the entire process of the biosynthesis and degradation of chlorophylls. PSI-475 upregulated genes encoding enzymes involved in chlorophyll production such as several isoforms of light-dependent protochlorophyllide oxidoreductases [*PORC* (*solyc07g054210.3.1*, FC +4.74); *PORA/B/C* (*solyc10g006900.3.1*, FC +3.41); *PORA/B* (*solyc12g013710.2.1*, FC +11.23)] or geranylgeranyl reductase [*ChlP* (*solyc03g115980.1.1*, FC +2.67)]. Moreover, genes encoding enzymes related to chlorophyll degradation such as chlorophyllase and magnesium dechelatase were also dysregulated [(*solyc06g053980.3.1*, FC +1.75); (*solyc09g065620.3.1,* FC +2.61); (solyc12g056480.2.1, FC −1.92)]. Interestingly, an upregulation of genes related to thiamine biosynthesis in both young leaf [*Thi4* (solyc07g064160.3.1, FC +18.85); *ThiC* (solyc06g006080.3.1; FC +2.24)] and root tissue [*Thi4* (solyc07g064160.3.1, FC +2.86); *ThiC* (solyc06g006080.3.1; FC +1.76)] was noted. 

**Carbohydrate metabolism:** 16 DEGs related to carbohydrate metabolism pathways were upregulated in young leaf tissue from treated plants with PSI-475, while 6 DEGs were downregulated. They include several genes involved in starch biosynthesis and degradation such as *AgpL1* (*solyc01g109790.3.1*, FC +2.27), *AgpL* (*solyc07g056140.3.1*, FC +1.89-fold), *GPI1* (*solyc04g076090.3.1*, FC +1.67) and beta amylase (*solyc01g067660.3.1*, FC −1.57) and one gene involved in sucrose metabolism [*SPS (solyc09g092130.3.1*, FC −5.11)]. Likewise, relevant genes related to trehalose-6-phosphate biosynthesis and degradation were upregulated [*TPS1* (*solyc07g062140.3.1*, FC +1.51)]; *TPPA* (*solyc04g054930.3.1*, FC +1.79)].

Regarding root tissue from treated plants, 25 and 26 genes were upregulated and downregulated compared to control, respectively. Similar to what was observed in young leaf tissue, several starch metabolism-related genes were dysregulated: [*AgpL1* (*solyc01g109790.3.1*, FC +4.18), *AgpL* (*solyc07g056140.3.1*, FC +2.74), beta amylase (*solyc08g077530.3.1*, FC −1.55), and beta amylase (*solyc09g091030.3.1*, FC −2.58)]. In contrast to what was observed in young leaf tissue, several members of the sucrose phosphate synthase gene family were strongly upregulated in root tissue [(*solyc09g092130.3.1*, FC +3.11); (*solyc07g007790.3.1*, FC +1.63), and (*solyc08g042000.3.1*, FC +1.61)]. Likewise, trehalose 6-phosphate phosphatase, which catalyses the conversion of trehalose-6-P to trehalose, was also strongly downregulated in root tissue [*TPPA* (*solyc03g083960.3.1*, FC −9.33)].

**Amino acid metabolism:** 23 genes were dysregulated in the amino acid and polyamine metabolism pathways of young leaf tissue, with 19 and 4 DEGs upregulated and downregulated, respectively. Two key genes related to proline metabolism were differentially expressed in treated stressed plants: while the transcript that encodes the enzyme that catalyses the terminal step in the biosynthesis of proline was downregulated [*P5CR* (*solyc02g068640.3.1*, FC −1.59)], a gene encoding an enzyme that catabolises proline was upregulated [*PDH* (*solyc02g089630.3.1*, FC +3.38)]. Moreover, DEGs related to the biosynthesis of folates and aromatic amino acids [*EPSP* (*solyc05g050980.3.1*, FC +1.81); *DAHP*, (*solyc04g074480.3.1*, FC +1.62)] and serine (*solyc06g076510.3.1*, FC +2.82) or gamma-aminobutyrate (GABA) metabolism [(*solyc01g005000.3.1*, FC +2.33); (*solyc12g006470.2.1*, FC +5.02)] were upregulated. Likewise, several genes related to polyamines such as spermidine and putrescine biosynthesis [(*solyc01g110430.3.1*, FC +1.71); *AdoMetDC* (*solyc01g010050.3.1*, FC +1.66)] and encoding the enzyme polyamine oxidase (PAO), which is known to regulate proline metabolism, were also upregulated [*PAO1* (*solyc01g087590.3.1*, FC +2.98)]. 

**Protein homeostasis, external stimuli response and redox homeostasis:** Identified genes in this category related to stress tolerance responses were a considerable number of heat shock proteins (HSPs). In young leaf and root tissue of treated plants, 26 and 22 DEGs were identified as *HSP90*, *HSP70*, *HSP40*, *HSP20*, and small *HSPs*, being upregulated in the majority. 

The chaperone *HSP101* (*solyc03g115230.3.1*, FC +2.47) and transcriptional regulator of HSPs *HsfA1* (*solyc08g005170.3.1*, FC +1.52) were found to be overexpressed in treated young leaf and root tissue, respectively. Moreover, the flowering time regulator GIGANTEA, which can interact with SOS2 kinase, was downregulated in treated root tissue (*solyc12g056650.2.1*, FC −1.81) but significantly upregulated in young leaf tissue of stressed plants treated with PSI-475 [(*solyc12g056650.2.1*, FC +6.43); (*solyc04g071990.3.1*, FC +2.64)].

Several DEGs involved in ROS detoxification were found to be dysregulated in young leaf and root tissue of treated plants. While 3 DEGs encoding antioxidant enzymes were upregulated in young leaf tissue [(*APX (solyc06g005150.3.1*, FC +1.89); *APX (solyc09g007270.3.1*, FC +2.54); M-type thioredoxin (*solyc01g108020.3.1*, FC +2.02)], a higher number of DEGs were overexpressed in root tissue (20 out of 37), including among them thioredoxins (*solyc05g006830.3.1*, FC +2.98), NADPH-oxidases (*solyc01g099620.3.1*, FC +1.85), SOD (*solyc02g021140.3.1*, FC +1.76) and CAT (*solyc02g082760.3.1*, FC +1.66).

**Not assigned:** Some important genes related to salinity stress tolerance were not assigned to any specific functional category by the MapMan software. Among them was the upregulation in treated leaf tissue of genes encoding LEA proteins such as *TAS14* (*solyc02g084850.3*, FC +1.69) or *LE25* (*solyc10g078770.2.1*, FC +1.51). 

### 2.10. Validation of RNA-Seq Data Using RT-qPCR

**Figure 6:**
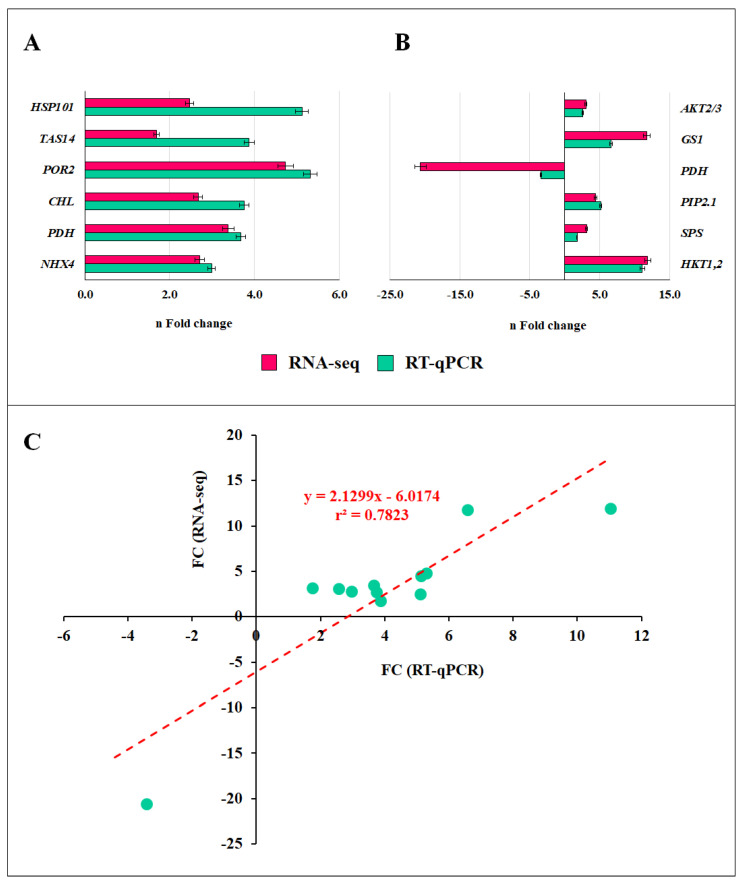
Validation of expression levels of DEGs obtained with RNA-seq using RT-qPCR. (**A**) FC comparative in young leaf tissue DEGs; (**B**) FC comparative in root tissue DEGs; (**C**) linear correlation between RT-qPCR and RNA-seq data.

In total, 11 DEGs involved in salinity stress tolerance mechanisms and belonging to the solute transport (*PIP2.1, AKT2/3, NHX4*, and *HKT1,2*), external stimuli (*HSP101, TAS14*), coenzyme metabolism (*POR2, CHLP*), amino acid metabolism (*GS1, PDH*) and carbohydrate metabolism (*SPS)* functional categories were selected for validation with RT-qPCR in young leaf and root tissues of salinity stressed treated plants (Figure 6). The RT-qPCR results showed that gene expression patterns were consistent with RNA-seq data in all selected DEGs from young leaf and root tissue. The correlation coefficient (r^2^) between RT-qPCR and FC values derived from RNA-seq was 0.782, indicating good reliability of the transcriptome data. 

## 3. Discussion

Salinity stress induces a series of responses in plants, including signal transduction, modulation of osmotic and ion homeostasis, activation of stress tolerance mechanisms and energy and substrate metabolic pathways [3,5,30,31]. An important objective of this study was integrating the data generated at four levels of biological information (phenotype, physiology, biochemistry, and transcriptome) in different plant tissues (root, lower stem, old leaf, upper steam, and young leaf) in a critical growth stage of tomato crop for crop productivity and quality (i.e., late fruit filling stage/early fruit harvest stage). While some authors have evaluated the effect of ANEs and PHBs in root and leaf tissues [27,32,33,34], the specific evaluation of metabolite partitioning in the root, stem and leaf tissues along with transcriptome studies in salinity stressed plants treated with PSI-475 brings a new level of detail on the mode of action of a biostimulant.

### 3.1. Impact of PSI-475 on Phenotypic and Yield Related Markers (Dose)

The plant experimental design used in this study aimed to apply a mild saline irrigation solution for several weeks (EC: 5.8 dS/m for 61 days), mimicking agronomic scenarios where growers do not have access to high quality water and need to use saline water for crop irrigation during long periods. The fact that the applied salinity stress had a statistically significant negative effect on phenotypic and yield parameters in untreated plants shows its suitability to challenge the bioactivity of PSI-475. 

A dosage effect was established for the biostimulant with the lowest PSI-475 dose applied (2.5 mL/L) in both growth conditions, providing similar or better effects than 6.25 and 12.5 mL/L, respectively (Table 1). This information is very relevant agronomically to establish the right balance between cost effectiveness per application in a high value vegetable crop and avoiding any overstimulating effect in the suggested application rate range. In fact, negative effects of high concentrations of various animal-derived PHBs applied by foliar spray and drench irrigation have been reported in tomato, maize and basil [33,35,36]. 

### 3.2. Effect of PSI-475 and Salinity Stress on Ion Homeostasis Metabolic Pathways

Untreated tomato plants irrigated with saline water accumulated higher Na^+^ content in upper stem and young leaf tissue, the opposite pattern was observed in those stressed plants treated with PSI-475, accumulating more Na^+^ in root and lower levels in stem tissue. This increase in Na^+^ in root tissue of treated plants was compensated with a higher K^+^ accumulation. Therefore, these results suggested that PSI-475 preferentially restricted Na^+^ translocation from roots to young leaves but promoted K^+^ accumulation in roots to cope with the harmful effects of Na^+^ accumulation in salinity stressed plants. It allowed the maintenance of higher K^+^/Na^+^ ratios in both young leaf and root tissue, which is known as a key predictor of salinity stress tolerance [37,38,39]. This finding can have very important implications in terms of ion toxicity avoidance promoted by PSI-475 because young leaf tissue from tomato plants is very sensitive to the harmful effects of Na^+^ accumulation [40]. Likewise, it has been observed that Na^+^ accumulation in roots of numerous woody species occurs as an adaptive response to prevent its toxicity in shoots and leaf organs [41,42]. As observed previously, different plant species also tend to protect young leaves from ion toxicity by deteriorating ion homeostasis in old leaves [43,44]. 

The comparative transcriptome analysis between PSI-475-treated and untreated young leaf and root tissue showed how multiple genes encoding solute transporters were dysregulated. HKT-1-like transporters have been proven to be important for preventing Na^+^ accumulation in aboveground photosynthetic tissues [45,46]. The strong upregulation of the *HKT1;2* gene in treated root tissue would suggest an increased Na^+^ removal from the xylem flow into xylem parenchymal cells, decreasing its translocation rate to young leaf tissue. Interestingly, similar results and conclusions were drawn by Almeida et al. (2014) [47] and Ali et al. (2018) [48]. As observed in our previous work [29], the application of PSI-475 induced the significant upregulation of a gene belonging to the vacuolar NHX cation-proton antiporter family (*NHX4*) in young leaf tissue, which could be associated with enhanced Na^+^ extrusion out of the cytoplasm. The overexpression of *NHX4* in salinity stressed transgenic tomato plants has been associated with improved stress tolerance mechanisms and enhanced fruit yield [49].

Overall, more genes closely related to K^+^ uptake and transport than those related to Na^+^ regulation were found to be upregulated. It suggests that the modulation of K^+^ content is as important as avoiding Na^+^ accumulation for maintaining a suitable K^+^/Na^+^ homeostasis in stressed plants treated with PSI-475. Given the enhanced K^+^ content in root tissue of treated plants (+79% with respect to stressed control), it can be speculated that the observed upregulation of *AKT2/3* in root tissue was involved in an optimised uptake of this cation [50]. Additionally, a concurrent upregulation of the TPK1 gene in both young leaf and root tissue of treated plants was observed. TPK channels are thought to play a role in plant cell K^+^ homeostasis by enabling regulated intracellular K^+^ transport from and into organelles, which could have a positive role in intercellular and intracellular K homeostasis [51]. The dysregulation of several genes encoding different cation/proton antiporters, such as CHX, was also observed. While some CHX genes have been proposed as candidates for pH homeostasis [52], CHX13, CHX17, CHX20, CHX21 and CHX23 have been shown to have a role in K^+^ accumulation and intracellular homeostasis [50,53]. The strong upregulation of the *CHX20* gene in root and young leaf tissue could be also associated with further benefits on osmotic stress tolerance by regulating osmoregulation of guard cells [53].

### 3.3. Effect of PSI-475 and Salinity Stress on Osmotic Adjustment Metabolic Pathways

One of the issues plants encounter when growing under salinity stress conditions is the loss of water because of decreased water conductivity in root tissue as Na^+^ accumulates in the soil [3,5]. Foliar application of PSI-475 on salinity stressed tomato plants resulted in an increase in RWC in young leaf tissue compared to untreated control. This benefit is of particular interest because reproductive stages are considered the most sensitive growth stage to drought stress in tomato growth [54]. This improvement in RWC may be linked to the significant presence in young leaf and root tissue of treated plants of DEGs encoding different aquaporins such as NIPs, PIPs, TIPs, XIPs and SIPs. According to a study developed by Li et al. (2016) [55], the application of drought or salinity stress in tomato plants (cv. Micro-Tom) caused a significant downregulation of three *PIP* genes in root tissue (*SlPIP2;1*, *SlPIP2;7* and *SlPIP2;5*). When the isolated *SlPIP2;1* gene was overexpressed in transgenic Arabidopsis and tomato lines, a significant increase in root water uptake and plant RWC under both unstressed and water stressed conditions was observed. Therefore, the marked upregulation of two DEG coding *PIP2.1* aquaporins in root tissue of tomato plants treated with PSI-475 could also be correlated to the increased RWC in young leaf tissue. Moreover, the upregulation of one *TIP2.1* gene in both young leaf and root tissue of treated plants could also be associated to an induced osmotic stress tolerance. As reported by Martins et al. (2017) [56], vacuolar *CsTIP2;1* aquaporin overexpressed in transgenic tobacco under unstressed, water and salinity-stressed conditions, resulted in a significant increase in plant growth and notable improvements in leaf water status and photosynthetic capacity.

As previously discussed by Ikuyinminu et al., 2022 [29], enhanced young leaf RWC in tomato plants treated with PSI-475 could also be explained by the accumulation of different organic osmolytes [5] including proline, a marker of salinity stress [57,58]. Proline accumulated in all untreated tissues from stressed tomato plants with a further increase in the content of this amino acid in young leaf and root tissue of treated plants. These changes were similar to those observed in salinity stressed lettuce and bean plants treated with plant-derived PHBs [59,60,61], or ANE in tomato [62,63]. Notably, proline concentrations were also reflected by the dysregulation of proline biosynthesis and conversion genes such as *PC5R, PHR1* and *PDH* [14,64,65]. Besides the protective role of proline during abiotic stress, it plays a key role for plant recovery during stress alleviation. Next to other osmolyte as GABA, proline may work as a ROS scavenger and source of N and C [63].

Salinity stress tolerance can also be assessed by changes in carbohydrate metabolism where some disaccharides, such as sucrose, accumulate and aid in maintaining membrane integrity and cell hydration levels [5,66]. Likewise, a further accumulation of soluble sugars in salinity stressed crops have been observed after the application of plant-derived PHBs [59,61]. The obtained metabolite and transcriptome data suggested different carbohydrate distribution patterns in young leaf and root tissue of stressed tomato plants treated with PSI-475. While sucrose content was highly accumulated in root tissue with respect to stressed control (+95%), only moderate increases of glucose and fructose were observed in the same tissue (+41% and +21%, respectively). However, an opposite pattern was observed in young leaf tissue, accumulating more glucose and fructose (+83% and +120%) than sucrose (+36%) with respect to the control. These differences on carbon partitioning to sucrose could have been influenced by the downregulation of one *SPS* gene in young leaf tissue and the significant upregulation of 3 *SPS* isoforms in root tissue [67]. Interestingly, a minor increase in glucose and fructose content and a decrease in sucrose content in treated old leaf tissue was measured, suggesting that carbohydrate translocation was mainly occurring between root and young leaf tissue through plant stems.

### 3.4. Effect of PSI-475 and Salinity Stress on Photosynthetic and N Metabolic Pathways

The reduction in photosynthetic pigments caused by increased salinity stress has been demonstrated in different studies [5,30,68,69]. Direct effects include controlling gene expression levels and activity of enzymes involved in chlorophyll biosynthesis and photosynthetic processes. Indirect effects are associated with the impact on the endogenous antioxidant system on photosynthetic processes [5,70]. As observed previously by Taffouo et al. (2010) [68] in five tomato cultivars, the application of a 61-day saline irrigation program using 50 mM NaCl was associated with a significant reduction in photosynthetic pigment content in tomato leaf organs. This reduction was more intense in older leaf tissues that displayed a chlorotic phenotype. Overall, the positive role of PSI-475 in increasing chlorophyll (a + b) and carotenoid content in young leaf tissue was in concordance with previous studies with salinity stressed plants treated with ANEs and PHBs [29,61,71].

The accumulation of chlorophyll (a + b) may be associated with the upregulation of DEGs encoding different enzymes involved in chlorophyll biosynthesis. Three protochlorophyllide oxidoreductases (POR) genes were strongly overexpressed in treated young leaf tissue (*PORC*, FC +4.74: *PORA/B/C*, FC +3.41; and *PORA/B* FC +11.23). Those are known to mediate the photoreduction of protochlorophyllide to chlorophyllide, which is then converted to chlorophyll in developing leaves [72]. The overexpression of *ChlP* geranylgeranyl reductase gene in young leaf tissue of treated plants was also reported to be associated with the higher chlorophyll content [73]. Finally, it is also important to mention that two genes related to chlorophyll degradation (e.g., chlorophyllase and magnesium dechelatase) were also dysregulated, which supports the model in which there is a balance between chlorophyll synthesis and breakdown, promoting its homeostasis during leaf growth [74].

The positive effect of PSI-475 on plant biomass and fruit yield in salinity stressed plants could also be explained by the significant dysregulation of DEGs involved in photosynthetic efficiency and amino acid metabolism. In this regard, PSI-475 application induced the upregulation of genes coding components of the photosynthetic electron transfer chain and enzymes involved in the process of CO_2_ fixation in the Calvin cycle. While previous studies have reported higher RuBisCo activity and overexpression of genes related to photosynthetic efficiency in unstressed maize and tomato treated with plant-derived PHBs [22,75], this is the first experimental evidence of such benefits in a vegetable crop subjected to long-term salinity stress. 

### 3.5. Effect of PSI-475 and Salinity Stress on Other Stress Response Related Pathways

The observed upregulation of genes encoding HSPs in tomato plants treated with PSI-475 could explain this effect at the metabolite level because these proteins can act as molecular chaperones that support folding of newly synthesized proteins or protect protein folding in stressful conditions [11,76]. While the upregulation of *HSPs* genes has also been observed in heat-stressed tomato plants treated with a specialized *Ascophyllum nodosum* extract (ANE) biostimulant [77] and unstressed tomato and maize plants after the application of PHBs [22,23], there are no studies that show similar molecular changes in salinity stressed plants. The significant overexpression of 2 DEGs encoding the LEA proteins TAS14 and LE25 could also suggest a positive role on maintenance of protein and membrane structures in salinity stressed plants treated with PSI-475. In a similar way, the application of specific ANEs have been associated with the simultaneous accumulation of TAS14 at the gene and protein level in leaf tissue of tomato plants with enhanced drought stress tolerance [78]. The positive role of LE25 protein has been also associated with improved cell protection from dehydration stress after being overproduced in *Saccharomyces cerevisiae* growing in a medium with 1.2 M NaCl [79].

### 3.6. Conclusions

**Figure 7:**
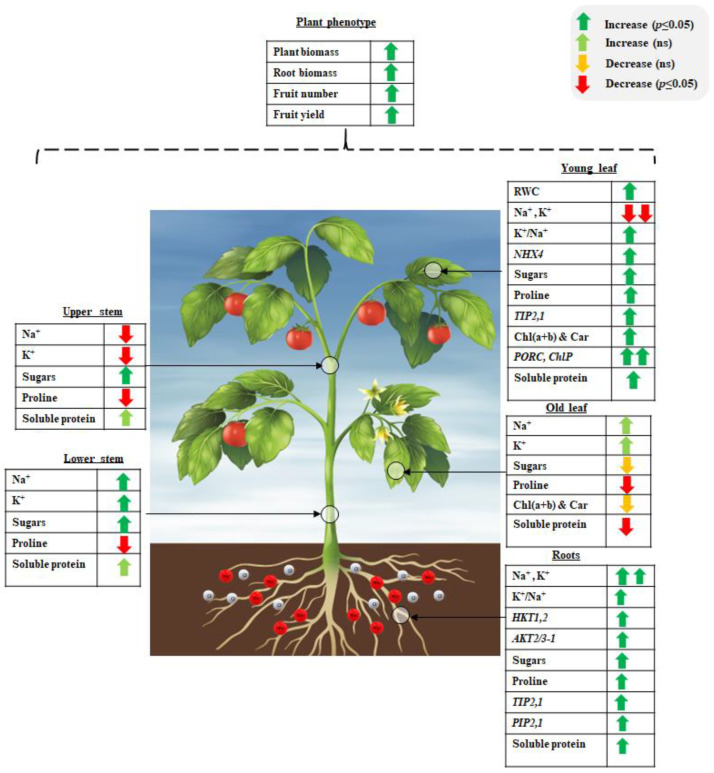
Diagram of PSI-475 mode of action inducing salinity stress tolerance in tomato after 61 days of saline water irrigation. Different parameters at phenotype level (root, plant and fruit) are shown along with physiological, biochemical, and molecular parameters in different plant tissues. Up or down arrow indicate increase of the parameter with respect to that obtained in untreated stressed plants. Different arrow colours indicate the outcome of the statistical hypothesis testing for that change: deep green and deep red imply statistically significant differences according to *t*-test at *p* ≤ 0.05, while orange and light green colours show non-significant changes at *p* ≤ 0.05.

Overall, this study found that PSI-475 produced a strong modification of transcriptional programming 34 days after the last foliar application and resulted in a significant upregulation of genes related to osmotic regulation, ion homeostasis, photosynthesis, and other metabolic pathways (Figure 7). Induced tissue partitioning of Na^+^ and K^+^ by PSI-475 was evident after measuring the ion content in root, stem, and leaf tissues, with a decreasing gradient between lower and upper plant organs. Young leaf tissue of treated plants was characterised by an improved osmotic stress tolerance at the physiological level (RWC) and was correlated with a higher content of osmolytes and overexpression of genes related to aquaporins. This plant organ, which is critical in providing photo-assimilates to developing fruits, also had an improved content of photosynthetic pigments and stimulated expression of genes involved in photosynthetic efficiency and chlorophyll biosynthesis. The ultimate impact of PSI-475 on salinity stress in agricultural practice will be judged by its effect on crop yield. In this study, PSI-475 increased yield in salinity stressed plants by 48% and closed the gap to within 3% of the yield of unstressed untreated plants. This performance clearly demonstrates that the molecular and physiological changes reported are significant for tomato production in salinity stressed environments.

## 4. Materials and Methods

### 4.1. Materials

The protein hydrolysate and A. nodosum-derived biostimulant (PSI-475) was provided by Brandon Bioscience (Tralee, Ireland) and its chemical and structural characterisation were described by [29]. Its composition includes ash (3.00 ± 0.15% *w*/*v*), total uronics (0.85 ± 0.07% *w*/*v*), fucose (0.81 ± 0.05% *w*/*v*), laminarin (0.29 ± 0.02% *w*/*v*), free mannitol (0.56 ± 0.06% *w*/*v*), polyphenols (1.22 ± 0.08% *w*/*v*), total free amino acids (1.90 ± 0.03% *w*/*v*) and soluble peptides (1.55 ± 0.02% *w*/*v*).

All chemical reagents and biochemical standards were purchased from Sigma-Aldrich (Arklow, Ireland) and Bio-Rad. The primers were purchased from Eurofins Genomics (Ebersberg, Germany).

### 4.2. Salinity Stress Tolerance Experimental Design in Tomato

**Figure 8:**
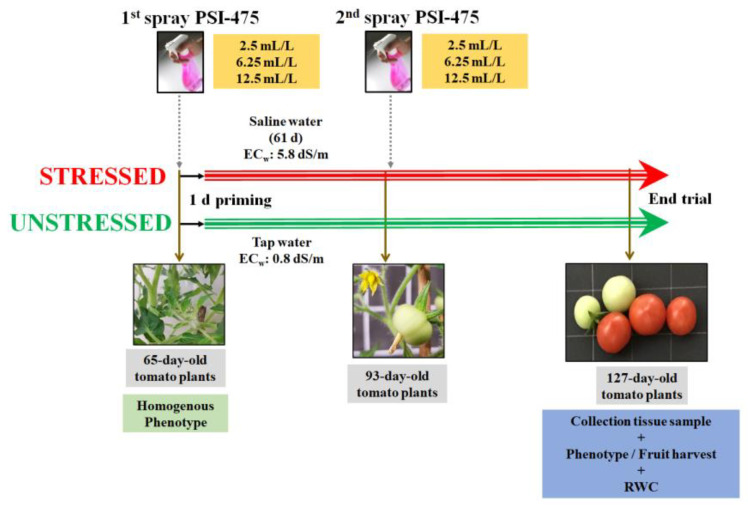
Graphical representation of tomato plant trials using a 61 day-saline irrigation water program to evaluate the bioactivity of PSI-475.

Tomato seeds (*S. Lycopersicum* L., cv. Micro Tom) were purchased from Moles Seeds (Essex, UK). After the germination of tomato seeds, transplant of obtained seedlings and fertilization program, the plants were grown in a growth room at a temperature of 27/22 ± 1 °C with 16 h of daylight, 8 h of night and 80 ± 5% RH under a light intensity of 120 μmol m^−2^ s^−1^ in a complete randomized block design. Prior to the application of salinity stress on the irrigation water, PSI-475 was applied at a rate of 2.5 mL/L, 6.25 mL/L and 12.5 mL/L by foliar spray to 65-day-old plants (early flowering stage). Water was applied as a control. After 1 day, salinity stress was started in half of the plants by watering them with 50 mM NaCl (EC: 5.8 dS/m). To minimize the influence of any positional effect, the relative position of the pots was changed every other day. Artificial pollination using an electric toothbrush was implemented once plants began to flower and was administered twice a week to promote cross pollination. After 27 days of adding saline water, the PSI-475 treatment was applied again as a foliar spray at the same rates described above (Figure 8). Control plants were sprayed with an equal volume of distilled water. Saline irrigation was maintained under conditions described above to obtain 127-day-old plants (late fruit filling stage/early fruit harvest stage). Phenotypic evaluation was performed at T0 (65-day-old plants) to confirm a homogenous growth stage for plants in the different groups before any treatment application. Additional phenotypical evaluation and tissue sampling was carried out at the end of plant trials (127-day-old plants) at 2 h after the end of the light period. After harvesting all fruits (ripe and unripe) for yield assessment, plants were divided into roots, lower stems (starting at the soil level and ending at the old leaves height), old leaves, upper stem (starting from old leaves and ending at young leaves height), young leaves and flowers. Root tissue was washed carefully with tap water to remove any growth medium particles. Then, every tissue sample of each 21 plants per treatment and condition was pooled, snap-frozen in liquid nitrogen, ground and kept in −80 °C until further analysis. The other half of the plants (control and treated with PSI-475) was grown under unstressed conditions for 127 days, watering them with tap water (0.73 dS/m). PSI-475 and control treatments were applied by foliar spray, as described above to evaluate their biostimulant effects on unstressed tomato plants.

### 4.3. Phenotypic Evaluation of Tomato Plants and Fruit Yield Assessment

Two phenotypic parameters were recorded at the end of the plant trials on 127-day-old-plants after dividing them into roots and aboveground plant organs. Plant biomass was determined after harvesting all the fruits. Root biomass was determined after drying the washed tissue for 12 h at 24 °C. Fruit number and yield was also measured, considering both ripe and unripe fruits.

### 4.4. Tomato Leaf RWC

RWC measurements were performed on young leaves collected at the end of plant according to [78]. Obtained leaf RWC was expressed on a % basis.

### 4.5. Biochemical Characterisation of the Collected Plant Tissues

Different metabolic markers were evaluated in root, lower stem, old leaf, upper stem and young leaf tissue of 127-day-old tomato plants untreated and treated with PSI-475 growing under unstressed and salinity stressed conditions. The content of Na^+^ and K^+^ in all the plant tissues were determined after their acid extraction according to [80]; proline content in all the plant tissues were evaluated as described by [78], a modified version of [81]; soluble sugars (as glucose, fructose and sucrose) in all the plant tissues were determined after extraction in water and PVPP; and soluble protein content in all the plant tissues were determined in all plant tissues indicated above as described by [78]. The photosynthetic pigments (chlorophyll a, chlorophyll b and carotenoids) from old and young leaf tissue were determined using an extraction method with water/acetone mixture according to [82]. Obtained data for all metabolites tested were expressed on a dry weight basis (mg/g DW)

### 4.6. RNA Extraction

RNA was extracted and purified from 30 mg of frozen ground young leaf and root tissue of 127-day-old tomato plants untreated and treated with PSI-475 applied at 2.5 mL/L growing under salinity stressed conditions (Figure S1) according to the manufacturer’s instructions of the Plant/Fungi Total RNA Purification Kit (Norgen Biotek Corp., Thorold, ON, Canada). RNA was treated with RNase-Free DNase I Kit (Norgen Biotek Corp., Thorold, ON, Canada) to remove efficiently genomic DNA contamination. 

### 4.7. RNA-Seq Analysis

Before proceeding to the cDNA library preparation, a quality control of RNA samples from young leaf and root tissue was performed. RNA degradation and contamination was monitored on 1% agarose gels. RNA purity was checked using the NanoPhotometer^®^ spectrophotometer (IMPLEN, Westlake Village, CA, USA). RNA integrity and quantification were also assessed using the RNA Nano 6000 Assay Kit of the Bioanalyzer 2100 system (Agilent Technologies, Santa Clara, CA, USA). Sequencing cDNA libraries were generated using NEBNext^®^ Ultra TM RNA Library Prep Kit for Illumina^®^ (NEB, Ipswich, MA, USA) following manufacturer’s recommendations and index codes were added to attribute sequences to each sample. The cDNA libraries quality was assessed on the Bioanalyzer 2100 system (Agilent Technologies, Santa Clara, CA, USA). The clustering of the index-coded samples was performed on a cBot Cluster Generation System using PE Cluster Kit cBot-HS (Illumina, San Diego, CA, USA) according to the manufacturer’s instructions. After cluster generation, the cDNA library preparations were sequenced on an Illumina Novaseq 6000 platform and paired-end reads were generated with an average read length of PE150.

Raw data (raw reads) of FASTQ format were firstly processed through *fastp*. All the downstream analyses were based on the clean data with high quality (Q20 and Q30 for PE150 reads ≥ 80%). Paired-end clean reads were mapped to the *Solanum lycopersicum* (tomato) genome assembly SL3.0 from Solanaceae Genomics Project (http://plants.ensembl.org/Solanum_lycopersicum/Info/Index (accessed on 12 September 2022)) using HISAT2 software. *Featurecounts* was used to count the read numbers mapped of each gene. Then, FPKM (fragments per kilobase of transcript per million mapped reads) of each gene was calculated based on the length of the gene and reads count mapped to this gene. 

Differential expression analysis between two conditions for each tissue (leaf treated vs. leaf untreated and root treated vs root untreated) was performed using *DESeq2* R package. The resulting *p* values were adjusted using the Benjamini and Hochberg’s approach for controlling the false discovery rate (FDR). Only changes in gene expression with an adjusted *p* ≤ 0.05 and a fold change (FC) (≥1.5 or ≤−1.5) among the three biological replicates were used to determine whether a gene was a DEG. DEG sets were selected for drawing Venn diagrams using a web-based tool (https://bioinformatics.psb.ugent.be/webtools/Venn/ (accessed on 15 September 2022)).

Gene Ontology (GO) enrichment analysis of obtained DEGs was performed using the *clusterProfiler* R package. GO terms with corrected *p* ≤ 0.05 were considered significantly enriched by DEGs. MapMan software (version 3.6.0RC1; https://mapman.gabipd.org/home (accessed on 19 September 2022)) was used to categorize and visualize obtained DEG sets on plant pathways with the most recent tomato genome annotation (ITAG4.0). Finally, the web-based AgriGO v2.0 gene ontology tool was also used to categorize those DEGs related to salinity stress tolerance mechanisms not assigned to any particular bin by MapMan software (http://systemsbiology.cau.edu.cn/agriGOv2/ (accessed on 22 September 2022)). For this purpose, the singular enrichment analysis (SEA) and the tomato genome annotation ITAG4.0 was used. 

### 4.8. Validation of RNA-Seq Analysis by qRT-PCR

qRT-PCR analysis using a Roche LightCycler^®^ 96 system was used to verify obtained RNA-seq results based on 11 selected genes related to salinity stress tolerance mechanisms. The primers of these genes are listed in Table S6 and the RNA samples used for qRT-PCR assays were the same as those used for RNA-seq. RNA concentration, purity and integrity were measured using Qubit (Thermo Fisher Scientific, Waltham, MA, USA). qRT-PCR assays were carried out using the same reaction conditions according to [29]. The expression level of the tomato TIP41 gene was used as a reference according to [83]. The 2^−ΔΔCT^ method was used to quantify relative normalized gene expression levels and they were compared to those from the same DEGs obtained in the RNA-seq analysis. Melting curves were used to evaluate the specificity of the amplification reaction. 

### 4.9. Statistical Analysis

Tomato plant and fruit phenotypic assessment was carried out in 3 independent trials with at least 21 plants per treatment and condition. For EC in tomato growth medium, RWC, biochemical, RNA-seq and qRT-PCR analysis, at least three biological replicates and three technical replicates per biological replicate were determined for each treatment and condition using the plant samples described above. Unless stated otherwise, all data are expressed as mean ± standard error (SE). Statistics were evaluated with Sigma Plot 12 and Statgraphics Centurion XVI software. Two-way ANOVA with Tukey’s HSD test (*p* ≤ 0.05) was used to compare the phenotypical, physiological, and biochemical data obtained from tomato. Where the interaction (S × P) between the two factors condition (S) and treatment (P) was significant, data were subjected to either *t*-test or one-way ANOVA by Tukey’s HSD test at *p* ≤ 0.05, comparing the treatment(s) versus the control within the same growth condition (unstressed or salinity stressed). The effect of condition and treatment was evaluated separately as well, comparing the respective means through either a *t*-test at *p* ≤ 0.05 or one-way ANOVA by Tukey’s HSD test at *p* ≤ 0.05. The application of all parametric tests was performed after checking the data normality (Shapiro–Wilk test) and equal variance assumptions.

## Figures and Tables

**Table 1:** Effect of salinity stress and PS1-475 dosage rate on tomato phenotypic and fruit yield parameters at the end of the plant trials.

Source of Variance	Plant Biomass (g)	Root Biomass (mg)	Fruit Number	Yield (g)
**Salinity (S)**				
Unstressed	18.02 b	127.88 b	9.07 b	12.86 b
Salinity stressed	13.81 a	108.71 a	7.89 a	9.47 b
**PSI-475 (P)**				
Untreated	15.05 a	74.57 a	7.31 a	8.45 a
PSI-475 2.5 mL/L	17.00 b	146.59 b	9.69 b	12.47 b
PSI-475 6.25 mL/L	16.96 b	136.50 b	8.57 b	12.35 b
PSI-475 12.5 mL/L	14.64 ab	115.52 b	8.36 ab	11.43 ab
**S × P**				
Unstressed × Untreated	17.52	76.02	8.00	9.47
Unstressed × PSI-475 2.5	19.63	171.92	10.24	13.91
Unstressed × PSI-475 6.25	18.26	143.59	8.57	14.67
Unstressed × PSI-475 12.5	16.66	119.99	9.48	13.41
Salinity stressed × Untreated	12.59	73.11	6.62	7.43
Salinity stressed × PSI-475 2.5	14.37	121.27	9.14	11.04
Salinity stressed × PSI-475 6.25	15.66	129.4	8.57	10.03
Salinity stressed × PSI-475 12.5	12.62	111.05	7.24	9.45
**Statistical significance**				
**Salinity (S)**	***	*	*	***
**PSI-475 (P)**	*	***	*	*
**S × P**	**ns**	**ns**	**ns**	**ns**

All data are expressed as average per plant. ns, *, ***: non-significant or significant at *p* ≤ 0.05, and *p* ≤ 0.001, respectively. Different letters within each parameter indicate statistically significant differences in the same factor according to *t*-test or one-way ANOVA by Tukey’s HSD test at *p* ≤ 0.05. Number of biological replicates (plant phenotypic and fruit yield markers: *n* ≥ 22).

**Table 2:** Effect of salinity stress and PSI-475 on sodium (Na^+^) content in different tomato plant tissues.

Source of Variance	Root (mg/g DW)	Lower Stem (mg/g DW)	Old Leaf (mg/g DW)	Upper Stem (mg/g DW)	Young Leaf (mg/g DW)
**Salinity (S)**					
Unstressed	31.21 a	9.90 a	3.46 a	10.16 a	1.43 a
Salinity stressed	73.11 b	91.78 b	66.19 b	90.09 b	70.26 b
**PSI-475 (P)**					
Untreated	49.41	45.93 a	34.30	55.11 b	41.46 b
PSI-475	59.92	55.75 b	35.35	45.14 a	30.23 a
**S × P**					
Unstressed × Untreated	35.73 a	10.55 a	3.29	10.67 a	2.05 a
Unstressed × PSI-475	26.70 a	9.25 a	3.64	9.65 a	0.80 a
Salinity stressed × Untreated	63.10 b	81.31 b	65.32	99.55 c	80.87 c
Salinity stressed × PSI-475	83.13 c	102.25 c	67.07	80.63 b	59.65 b
**Statistical significance**					
**Salinity (S)**	***	***	***	***	***
**PSI-475 (P)**	**ns**	**	**ns**	***	***
**S × P**	***	***	**ns**	***	***

All data are expressed as average per sample collected at harvest sample points. ns, **, and *** means non-significant or significant at *p* ≤ 0.01, and *p* ≤ 0.001, respectively. Different letters indicate statistical differences with *p* ≤ 0.05 based on *t*-test (S, P) or Tukey’s HSD test (S × P). Number of biological replicates (*n* ≥ 3).

**Table 3:** Effect of salinity stress and PSI-475 on potassium (K^+^) content in different tomato plant tissues.

Source of Variance	Root (mg/g DW)	Lower Stem (mg/g DW)	Old Leaf (mg/g DW)	Upper Stem (mg/g DW)	Young Leaf (mg/g DW)
**Salinity (S)**					
Unstressed	48.01 b	290.23 b	134.12 b	240.02	101.66 b
Salinity stressed	37.35 a	259.41 a	115.78 a	199.26	91.70 a
**PSI-475 (P)**					
Untreated	27.78 a	280.05	124.17	231.47 b	101.45 b
PSI-475	57.58 b	269.59	125.73	207.81 a	91.91 a
**S × P**					
Unstressed × Untreated	28.79 a	314.12 c	136.02	242.24 b	104.84
Unstressed × PSI-475	62.24 c	266.35 ab	132.23	237.79 b	98.48
Salinity stressed × Untreated	26.77 a	245.98 a	112.32	220.70 b	98.06
Salinity stressed × PSI-475	47.92 b	272.84 b	119.23	117.83 a	85.34
**Statistical significance**					
**Salinity (S)**	***	***	***	***	***
**PSI-475 (P)**	***	**ns**	**ns**	***	***
**S × P**	***	***	**ns**	**	**ns**

All data are expressed as average per sample collected at harvest sample points. ns, ** and *** means non-significant or significant at *p* ≤ 0.01 and *p* ≤ 0.001, respectively. Different letters indicate statistical differences with *p* ≤ 0.05 based on *t*-test (S, P) or Tukey’s HSD test (S × P). Number of biological replicates (*n* ≥ 3).

**Table 4:** Effect of salinity stress and PSI-475 on proline content in different tomato plant tissues.

Source of Variance	Root (mg/g DW)	Lower Stem (mg/g DW)	Old Leaf (mg/g DW)	Upper Stem (mg/g DW)	Young Leaf (mg/g DW)
**Salinity (S)**					
Unstressed	1.12 a	1.07 a	1.43 a	0.67 a	1.01 a
Salinity stressed	2.24 b	6.05 b	12.72 b	3.76 b	13.21 b
**PSI-475 (P)**					
Untreated	1.58 a	3.84 b	7.51 b	2.59 b	6.86 a
PSI-475	1.78 b	3.29 a	6.64 a	1.83 a	7.36 b
**S × P**					
Unstressed × Untreated	1.05	1.05 a	1.55 a	0.72 a	1.05 a
Unstressed × PSI-475	1.19	1.10 a	1.31 a	0.61 a	0.97 a
Salinity stressed × Untreated	2.11	6.63 c	13.48 c	4.46 c	12.67 b
Salinity stressed × PSI-475	2.37	5.48 b	11.96 b	3.06 b	13.75 c
**Statistical significance**					
**Salinity (S)**	***	***	***	***	***
**PSI-475 (P)**	***	***	***	***	***
**S × P**	**ns**	***	***	***	***

All data are expressed as average per sample collected at harvest sample points. ns and *** means non-significant or significant at *p* ≤ 0.001. Different letters indicate statistical differences with *p* ≤ 0.05 based on *t*-test (S, P) or Tukey’s HSD test (S × P). Number of biological replicates (*n* ≥ 3).

**Table 5:** Effect of salinity stress and PSI-475 on sucrose content in different tomato plant tissues.

Source of Variance	Root (mg/g DW)	Lower Stem (mg/g DW)	Old Leaf (mg/g DW)	Upper Stem (mg/g DW)	Young Leaf (mg/g DW)
**Salinity (S)**					
Unstressed	1.95 a	3.07	1.30 a	3.22	1.12 a
Salinity stressed	3.47 b	2.65	2.27 b	3.23	2.45 b
**PSI-475 (P)**					
Untreated	1.87 a	2.16 a	1.91 a	2.93 a	1.56 a
PSI-475	3.55 b	3.56 b	1.66 a	3.51 b	2.01 b
**S × P**					
Unstressed × Untreated	1.39 a	2.42	1.33	2.93	0.99
Unstressed × PSI-475	2.52 b	3.72	1.28	3.50	1.25
Salinity stressed × Untreated	2.35 b	1.91	2.50	2.94	2.12
Salinity stressed × PSI-475	4.59 c	3.40	2.04	3.52	2.77
**Statistical significance**					
**Salinity (S)**	***	**ns**	**	**ns**	***
**PSI-475 (P)**	***	***	*	**	**
**S × P**	***	**ns**	**ns**	**ns**	**ns**

All data are expressed as average per sample collected at harvest sample points. ns, *, ** and *** means non-significant or significant at *p* ≤ 0.05, *p* ≤ 0.01 and *p* ≤ 0.001, respectively. Different letters indicate statistical differences with *p* ≤ 0.05 based on *t*-test (S, P) or Tukey’s HSD test (S × P). Number of biological replicates (*n* ≥ 3).

**Table 6:** Effect of salinity stress and PSI-475 on photosynthetic pigments content in tomato leaf tissues.

Source of Variance	Old Leaf Chl (a + b) (mg/g DW)	Old Leaf Carotenoids (mg/g DW)	Young Leaf Chl (a + b) (mg/g DW)	Young Leaf Carotenoids (mg/g DW)
**Salinity (S)**				
Unstressed	10.16 b	1.75 b	13.27 b	2.31 b
Salinity stressed	6.48 a	1.19 a	10.85 a	1.94 a
**PSI-475 (P)**				
Untreated	8.55 b	1.46	11.73	2.07
PSI-475	8.09 a	1.48	12.39	2.18
**S × P**				
Unstressed × Untreated	10.50	1.76	13.91 c	2.37 b
Unstressed × PSI-475	9.83	1.74	12.63 b	2.26 b
Salinity stressed × Untreated	6.60	1.16	9.54 a	1.76 a
Salinity stressed × PSI-475	6.36	1.22	12.16 b	2.11 b
**Statistical significance**				
**Salinity (S)**	***	***	***	***
**PSI-475 (P)**	*	**ns**	**ns**	**ns**
**S × P**	**ns**	**ns**	***	**

All data are expressed as average per sample collected at harvest sample points. ns, *, ** and *** means non-significant or significant at *p* ≤ 0.05, *p* ≤ 0.01 and *p* ≤ 0.001, respectively. Different letters indicate statistical differences with *p* ≤ 0.05 based on *t*-test (S, P) or Tukey’s HSD test (S × P). Number of biological replicates (*n* ≥ 3).

## Data Availability

The complete raw RNAseq data from this publication have been submitted to the GEO database (http://www.ncbi.nlm.nih.gov/geo/ (accessed on 16 November 2022)) and assigned the identifier accession PRJNA899854.

## Supplementary Materials

The supporting information can be downloaded at: https://www.mdpi.com/article/10.3390/ijms24086988/s1.
