# Supplementary material for: Transcriptome, Biochemical and Phenotypic Analysis of the Effects of a Precision Engineered Biostimulant for Inducing Salinity Stress Tolerance in Tomato
Source: Int J Mol Sci. 2023 Apr 10;24(8):6988. doi: 10.3390/ijms24086988 (PMC10138596; doi:10.3390/ijms24086988)
Supplement: Supplementary file 1 [file ijms-24-06988-s001.zip › ijms-2280794-supplementary.pdf]

International Journal of *Molecular Sciences*

MDPI

# Transcriptome, Biochemical and Phenotypic Analysis of the Effects of a Precision Engineered Biostimulant for Inducing Salinity Stress Tolerance in Tomato

**Elomofe Ikuyinminu [1,2], Oscar Goñi [1,2,*], Łukasz Łangowski [2] and Shane O'Connell [1,2]**

[1] Plant Biostimulant Group, Shannon Applied Biotechnology Centre, Munster Technological University-Tralee (South Campus), Clash, V92 CX88 Tralee, Co. Kerry, Ireland
[2] Brandon Bioscience, V92 N6C8 Tralee, Co. Kerry, Ireland
* Correspondence: oscar.goni@mtu.ie

## Supplementary Material

### 1 Supplementary Figures

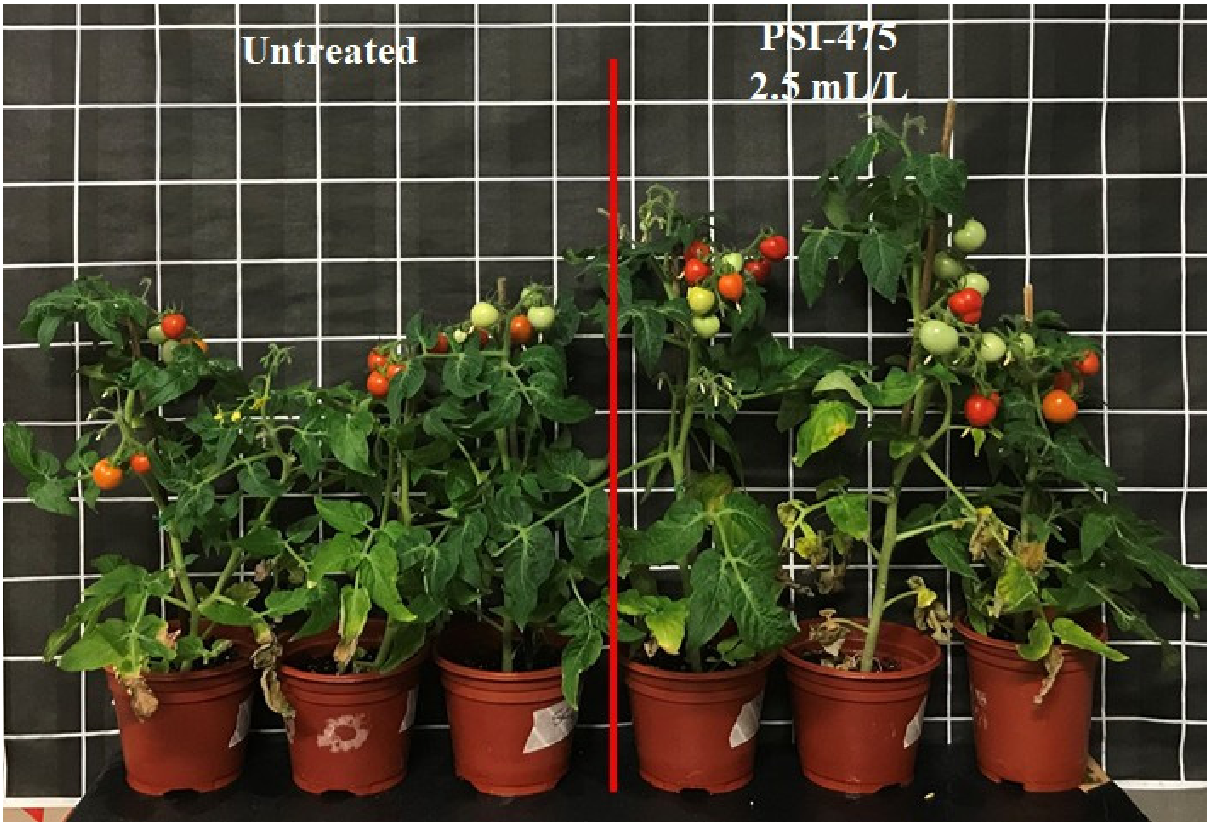


**Figure S1.** Pictures of the plants used to generate the transcriptomic data. Scale of background square equals 5 cm H X by 5 cm W.

## 2 Supplementary Tables

**Table S1.** Effect of salinity stress and PSI-475 on $K^+/Na^+$ ratio in different tomato plant tissues.

| Source of variance | Root | Lower stem | Old leaf | Upper stem | Young leaf |
|---|---|---|---|---|---|
| **Salinity (S)** | | | | | |
| Unstressed | 1.66 b | 30.60 b | 36.24 b | 24.01 b | 102.73 b |
| Salinity stressed | 0.51 a | 2.86 a | 1.76a | 2.21 a | 1.32 a |
| **PSI-475 (P)** | | | | | |
| Untreated | 0.62 a | 16.25 | 19.60 b | 12.52 | 32.87 a |
| PSI-475 | 1.54 b | 17.21 | 18.40 a | 13.70 | 71.19 b |
| **S × P** | | | | | |
| Unstressed ×Untreated | 0.81 c | 29.46 | 37.46 c | 22.82 | 65.52 b |
| Unstressed × PSI-475 | 2.51 d | 31.75 | 35.03 b | 25.19 | 140.94 c |
| Salinity stressed × Untreated | 0.43 a | 3.05 | 1.74 a | 2.21 | 1.22 a |
| Salinity stressed × PSI-475 | 0.58 b | 2.67 | 1.78 a | 2.21 | 1.43 a |
| **Statistical significance** | | | | | |
| **Salinity (S)** | *** | *** | *** | *** | *** |
| **PSI-475 (P)** | *** | **ns** | *** | **ns** | * |
| **S × P** | *** | **ns** | *** | **ns** | * |

All data are expressed as average per sample collected at harvest sample points. ns, *, and *** means non-significant or significant at $p \leq 0.05$, and $p \leq 0.001$, respectively. Different letters indicate statistical differences with $p \leq 0.05$ based on t-test (S, P) or Tukey's HSD test (S × P). Number of biological replicates ($n \geq 3$).

**Table S2.** Effect of salinity stress and PSI-475 on glucose content in different tomato plant tissues.

| Source of variance | Root (mg/g DW) | Lower stem (mg/g DW) | Old leaf (mg/g DW) | Upper stem (mg/g DW) | Young leaf (mg/g DW) |
|---|---|---|---|---|---|
| **Salinity (S)** | | | | | |
| Unstressed | 0.24 | 0.79 b | 0.68 a | 1.43 a | 0.80 a |
| Salinity stressed | 0.30 | 0.56 a | 2.06 b | 0.73 b | 1.78 b |
| **PSI-475 (P)** | | | | | |
| Untreated | 0.21 a | 0.32 a | 1.20 a | 0.71 a | 0.98 a |
| PSI-475 | 0.33 b | 1.04 b | 1.55 b | 1.45 b | 1.60 b |
| **S × P** | | | | | |
| Unstressed × Untreated | 0.16 | 0.24 a | 0.54 | 0.48 a | 0.70 |
| Unstressed × PSI-475 | 0.32 | 1.35 c | 0.83 | 2.37 b | 0.90 |
| Salinity stressed × Untreated | 0.25 | 0.40 a | 1.86 | 0.93 a | 1.26 |
| Salinity stressed × PSI-475 | 0.34 | 0.73 b | 2.26 | 0.53 a | 2.31 |
| **Statistical significance** | | | | | |
| **Salinity (S)** | ns | * | *** | ** | *** |
| **PSI-475 (P)** | * | *** | *** | ** | * |
| **S × P** | ns | *** | ns | *** | ns |

All data are expressed as average per sample collected at harvest sample points. ns, *, **, and *** means non-significant or significant at $p \leq 0.05$, $p \leq 0.01$, and $p \leq 0.001$, respectively. Different letters indicate statistical differences with $p \leq 0.05$ based on t- test (S, P) or Tukey's HSD test (S × P). Number of biological replicates ($n \geq 3$).

**Table S3.** Effect of salinity stress and PSI-475 on fructose content in different tomato plant tissues.

| Source of variance | Root (mg/g DW) | Lower stem (mg/g DW) | Old leaf (mg/g DW) | Upper stem (mg/g DW) | Young leaf (mg/g DW) |
|---|---|---|---|---|---|
| **Salinity (S)** | | | | | |
| Unstressed | 0.50 | 0.75 | 1.01 a | 1.36 b | 1.25 a |
| Salinity stressed | 0.47 | 0.89 | 4.76 b | 0.87 a | 2.45 b |
| **PSI-475 (P)** | | | | | |
| Untreated | 0.35 a | 0.50 a | 2.63 | 0.69 a | 1.17 a |
| PSI-475 | 0.62 b | 1.14 b | 3.15 | 1.54 b | 2.53 b |
| **S × P** | | | | | |
| Unstressed × Untreated | 0.27 a | 0.26 a | 0.73 | 0.46 a | 0.82 a |
| Unstressed × PSI-475 | 0.72 b | 1.24 c | 1.29 | 2.26 c | 1.68 b |
| Salinity stressed × Untreated | 0.42 a | 0.74 b | 4.52 | 0.91 b | 1.53 ab |
| Salinity stressed × PSI-475 | 0.51 ab | 1.04 bc | 5.00 | 0.82 b | 3.38 c |
| | | **Statistical significance** | | | |
| **Salinity (S)** | **ns** | **ns** | *** | *** | *** |
| **PSI-475 (P)** | ** | *** | **ns** | *** | *** |
| **S × P** | * | ** | **ns** | *** | * |

All data are expressed as average per sample collected at harvest sample points. ns, *, **, and *** means non-significant or significant at $p \leq 0.05$, $p \leq 0.01$, and $p \leq 0.001$, respectively. Different letters indicate statistical differences with $p \leq 0.05$ based on t- test (S, P) or Tukey's HSD test (S × P). Number of biological replicates ($n \geq 3$).

**Table S4.** Effect of salinity stress and PSI-475 on soluble protein content in different tomato plant tissues.

| Source of variance | Root (mg/g DW) | Lower stem (mg/g DW) | Old leaf (mg/g DW) | Upper stem (mg/g DW) | Young leaf (mg/g DW) |
|---|---|---|---|---|---|
| **Salinity (S)** | | | | | |
| Unstressed | 17.11 b | 8.24 | 20.52 b | 10.60 | 43.91 |
| Salinity stressed | 10.76 a | 7.79 | 14.01 a | 9.02 | 42.44 |
| **PSI-475 (P)** | | | | | |
| Untreated | 13.33 | 7.44 | 18.93 b | 9.60 | 39.80 a |
| PSI-475 | 14.55 | 8.60 | 15.61 a | 10.03 | 46.54 b |
| **S × P** | | | | | |
| Unstressed × Untreated | 16.87 | 7.31 | 20.04 c | 10.99 | 41.61 |
| Unstressed × PSI-475 | 17.37 | 9.18 | 21.01 c | 10.22 | 46.20 |
| Salinity stressed × Untreated | 9.79 | 7.58 | 17.82 b | 8.21 | 37.99 |
| Salinity stressed × PSI-475 | 11.73 | 8.01 | 10.21 a | 9.36 | 46.88 |
| | | Statistical significance | | | |
| **Salinity (S)** | *** | **ns** | *** | **ns** | **ns** |
| **PSI-475 (P)** | **ns** | **ns** | *** | **ns** | *** |
| **S × P** | **ns** | **ns** | *** | **ns** | **ns** |

All data are expressed as average per sample collected at harvest sample points. ns and *** means non-significant or significant $p \leq 0.001$. Different letters indicate statistical differences with $p \leq 0.05$ based on t- test (S, P) or Tukey's HSD test (S × P). Number of biological replicates ($n \geq 3$).

**Table S5.** RNA-seq QC data summary in young leaf and root tissue samples of salinity stressed tomato plants.

| Sample name[1] | Raw reads[2] | Clean reads[3] | Error rate (%)[4] | Q20 (%)[5] | Q30 (%)[6] | GC content (%)[7] |
|---|---|---|---|---|---|---|
| LeafTrt2 | 28,233,239 | 27,758,977 | 0.02 | 98.56 | 95.47 | 42.79 |
| LeafTrt3 | 25,557,968 | 25,230,307 | 0.02 | 98.66 | 95.70 | 42.74 |
| LeafTrt1 | 27,191,658 | 26,799,960 | 0.02 | 98.68 | 95.75 | 43.06 |
| RootTrt2 | 21,395,116 | 20,865,057 | 0.02 | 98.65 | 95.64 | 42.34 |
| RootTrt3 | 25,815,340 | 25,134,755 | 0.02 | 98.52 | 95.32 | 42.48 |
| RootTrt1 | 22,280,918 | 21,782,527 | 0.02 | 98.61 | 95.50 | 42.34 |
| LeafCtr3 | 23,909,857 | 23,588,287 | 0.02 | 98.64 | 95.67 | 42.86 |
| LeafCtr2 | 22,323,457 | 21,922,394 | 0.02 | 98.54 | 95.42 | 42.65 |
| LeafCtr1 | 24,066,624 | 23,750,535 | 0.02 | 98.56 | 95.49 | 42.58 |
| RootCtr3 | 23,636,644 | 23,136,802 | 0.02 | 98.62 | 95.58 | 42.20 |
| RootCtr2 | 23,0231,55 | 22,730,435 | 0.02 | 98.51 | 95.32 | 42.17 |
| RootCtr1 | 21,560,363 | 21,210,996 | 0.02 | 98.50 | 95.27 | 42.14 |

[1] "Trt" and "Ctr" correspond to treated with PSI-475 and control samples.
[2] Reads count from the raw data, four rows as a unit, with statistics of reads count for every sequencing.
[3] Reads count filtered from raw data. Statistics method is similar with raw reads. All the following analysis is based on clean data.
[4] Base error rate of whole sequencing.
[5] The percentage of the bases whose Q Phred values is greater than 20 (99% correct rate).
[6] The percentage of the bases whose Q Phred values is greater than 30 (99.9% correct rate).
[7] The percentage of G&C base numbers of total bases.

**Table S6.** Primers sequences used for qRT-PCR analysis in tomato

| Gene | Primer Forward | Primer Reverse |
|---|---|---|
| *TIP41 (Solyc07g025390.4)* | CTCGACCAAGTCCCTACGAT | TGAAAAGCTTGCGACACTCG |
| *NHX4 (Solyc01g098190.3)* | TGGGAGCAATTTCACGACGA | TTCCAGCGCCTTTCACCAAT |
| *SPS (Solyc09g092130.3.1)* | AAAACGCCGTCAAGAACGTG | GCAATCGGCCTCTGGTACTT |
| *PDH (Solyc02g089630.3.1)* | GGTTTTTCGTGACCTCCGAC | AGAACCATAGTTGCCGGTGA |
| *CHL (Solyc03g115980.1)* | TGGCCAACATACAAGGTGCT | AATGGGGTTTCCTGGTGCAA |
| *TAS14 (Solyc02g084850.3)* | TGCCTGGACAACATGAAGGT | GCGTCAGCACACTTTACACG |
| *HSP101 (Solyc03g115230.3.1)* | ACCCGATCAGATTGCGGAAG | GAACCAGTTGGTTGCTGTGG |
| *GS1 (Solyc04g014510.3.1)* | ATCGCGGAGACTACCATCCT | AGGTCATGTGAGGAAAGGGC |
| *AKT2/3 (Solyc01g104030.3.1)* | CGGAGACGCTCAAGGAAGAA | TGCATCCATGTCTAAGAAGCAC |
| *PIP2.1 (Solyc10g084120.2.1)* | CAGCTGGCATTTCTGGTGGA | CAGCACCTAATCCAGTGGCTA |
| *POR2 (Solyc07g054210.3.1)* | GCTGCATTGCTGAAACTGGT | TCTCTGCATCACTGGCTTCC |
| *HKT1;2 (Solyc07g014680.3)* | CCTACCGTCTTTTCGTCCTCA | AGGTAAAAGCTTCCCCACCA |
